# Nonlinear Distortion by Stimulated Brillouin Scattering in Kramers-Kronig Receiver Based Optical Transmission

**DOI:** 10.3390/s22197287

**Published:** 2022-09-26

**Authors:** Yuzhu Zhu, Jiangbing Du, Weihong Shen, Zuyuan He

**Affiliations:** 1 AVIC Aeronautical Radio Electronics Research Institute, Shanghai 200241, China; 2 State Key Laboratory of Advanced Optical Communication Systems and Networks, Shanghai Jiao Tong University, Shanghai 200240, China

**Keywords:** nonlinear distortion, Kramers–Kronig receiver, stimulated Brillouin scattering

## Abstract

Nonlinear distortion for single-sideband (SSB) signals will significantly reduce the performance of Kramers–Kronig (KK) receiver-based optical transmission. In this work, we present a proof-of-concept study of stimulated Brillouin scattering (SBS)-induced nonlinear distortion for 10 Gbaud and 28 Gbaud SSB QAM16 transmission over 80 km standard single mode fiber (SSMF) based on a KK receiver. Significantly reduced bit error rate (BER) has been experimentally observed due to the SBS and the threshold of SBS at about 7 dBm is detected for such an 80 km SSMF link. With left sideband (LSB) modulation of SSB, together with optical filtering, reduced SBS nonlinear distortion has been achieved with ~2 dB power tolerance improvement. The results reveal an important issue of SBS-induced nonlinear distortion, which would be of great significance for KK receiver-based optical transmission applications.

## 1. Introduction

In recent years, the rapid development of information industries, such as autonomous driving, 5G, and so on, has led to an urgent demand for data transmission, especially for optical interconnection. Intensity modulation/direct detection (IM/DD) is widely used for optical interconnection at conventional 1310 nm and 15,500 nm wavebands, and has recently been extended to the 2 micron waveband [1,2]. Advanced modulations have been proposed for versatile IM/DD systems [3,4,5], in which nonlinear distortion becomes a very fatal issue due to the much tighter signal-to-noise ratio (SNR) budget, as well as the higher peak-to-average-power-ratio (PAPR). Normally, DD system can only be applied to IM signal for the detection of amplitude information rather than phase information [6]. In 2016, Mecozzi et al. proposed a Kramers–Kronig (KK) self-coherent receiver [7]. On the basis of satisfying the minimum phase condition of signal [8], the phase of the optical field can be restored by its intensity through a KK relationship by using a single photodetector (PD) [9,10,11,12]. This scheme has attracted widespread attention since it was proposed. Many studies have been carried out which prove the excellent performance of the KK receiver [13,14,15].

However, the KK receiver requires that the signal should meet the minimum phase condition, leading to a single sideband modulation (SSB) with a considerably high carrier-to-signal power ratio (CSPR), which could be over 10 dB. Serious nonlinear distortion can be expected for such a SSB signal due to the high CSPR, high PAPR, and tight SNR budget. The high CSPR is needed to maintain a small bit error rate (BER) [16,17,18]. However, on the other hand, nonlinear distortion by stimulated Brillouin scattering (SBS) will be easily excited for km-level distance optical fiber transmission. Particularly, with a guard band between the carrier and signal, the carrier itself can be considered as a standalone narrow linewidth continuous wave, which makes the SBS threshold even smaller, leading to worse SBS nonlinear distortion. Therefore, the investigation of SBS based nonlinear distortion to KK receiver-based optical fiber transmission is urgently needed.

In this work, we carried out a proof-of-concept study with solid experiments to unfold the nonlinear distortion limitation by SBS for KK signals, which is a unique phenomenon for KK receiver due to the high CSPR property of SSB signal. A significant reduction in the transmission performance due to SBS is observed for 10 Gbaud and 28 Gbaud SSB QAM16 signal transmissions over 80 km SSMF based on a KK receiver. The method of sideband filtering is proposed to reduce nonlinear distortion by SBS. Those results are of increasing value to researchers in this field, along with the increased use of a KK receiver in optical communication applications, which offer a higher data rate and long transmission distance.

## 2. Nonlinear Distortion Due to SBS for SSB Signals

**Figure 1:**
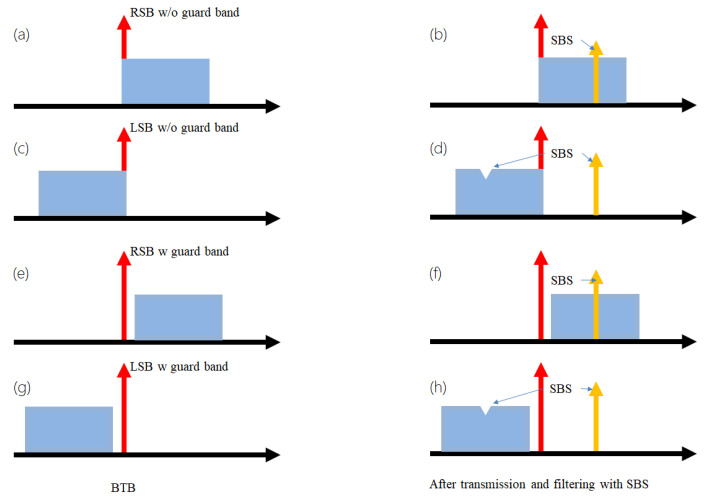
SBS induced nonlinear distortion for SSB signals. (**a**) RSB without guard band; (**b**) RSB without guard band after SBS distortion; (**c**) LSB without guard band; (**d**) LSB without guard band after SBS distortion; (**e**) RSB with guard band; (**f**) RSB with guard band after SBS distortion; (**g**) LSB with guard band; (**h**) LSB with guard band after SBS distortion.

Figure 1 shows the schematic mechanism of SBS induced nonlinear distortion for SSB signals. The generation of a SSB signal can be realized by standard in-phase and quadrature (IQ) modulation, optical filtering, or carrier-signal offset combination, which is adopted in this work. As depicted by Figure 1a,c, left single-sideband (LSB) and right single-sideband (RSB) SSB signals can be flexibly obtained. The SSB signal can be realized with or without guard band between the carrier and the signal. Improved optical detection can be obtained for SSB with guard band, as shown by Figure 1e,g.

As an important nonlinear distortion, SBS can be easily induced due to its comparably low threshold. In conventional applications, the signals are modulated with a high data rate without significant carrier power left and, thus, lead to neglected SBS. However, for the KK receiver, the SSB signal is needed with a high carrier power in order to maintain a small detection error. Typical CSPR exceeds 10 dB, which makes the SBS issue no longer neglectable, as shown in Figure 1b,d. In particular, as for SSB signals with guard band, the carrier is in fact a standalone laser with narrow linewidth. The SBS threshold would be even lower, and significant nonlinear distortion due to SBS can be expected, as schematically shown in Figure 1f,h.

## 3. Experimental Setup

**Figure 2:**
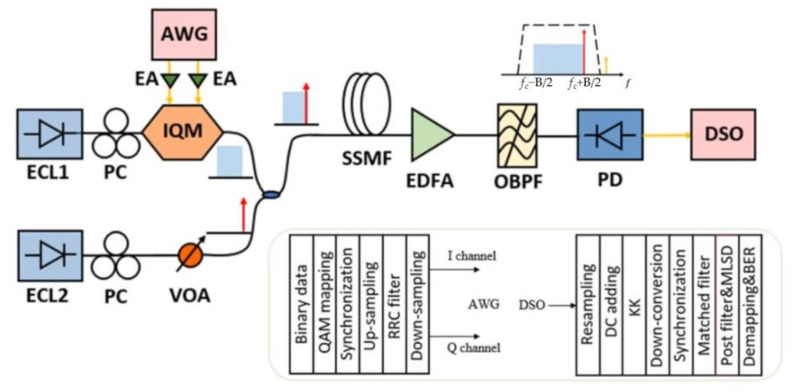
Experiment setup and DSP. Here, ECL: external cavity laser; PC: polarization controller; IQM: in-phase and quadrature modulator; AWG: arbitrary waveform generator; VOA: variable optical attenuator; EDFA: erbium-doped fiber amplifier; OBPF: optical bandpass filter; DSO: digital storage oscilloscope.

The experimental setup for the KK receiver-based optical transmission is built for the investigation of the SBS induced nonlinear distortion for SSB signals, as shown in Figure 2.

At the transmitter, a pseudo-random bit stream (2^17^−1) is generated by a 54 GSa/s arbitrary waveform modulator (AWG), which is mapped into a QAM16 signal after encoding. The baud rate $R_{s}$ is set to 10 Gbaud and 28 Gbaud, respectively. Then, the signal is pulse-shaped with a root-raised cosine (RRC), and the roll-off coefficient α=0.01. The signal spectrum bandwidth is $B_{s}=\left(1+\alpha \right)R_{s}$. A continuous wave light from an external cavity laser (ECL1) at 1550 nm is fed into the IQ modulator. Additionally, the other ECL (ECL2, with a linewidth of 15 KHz) generates a continuous wave (CW) as an optical carrier, whose power can be adjusted to obtain a different output total power and CSPR. The optical SSB QAM16 signal is then launched into the 80 km standard single mode-fiber (SSMF) at 1550 nm.

At the receiver, the transmitted optical field is first detected by the PD with a 3 dB bandwidth of ~22 GHz, and the electrical signal is captured by an 80 GSa/s digital storage oscilloscope (DSO, Keysight DSOZ592A). As shown by the inset of Figure 2, an optical bandpass filter (OBPF) before PD can be used for the filtering of the SBS influence, so as to reduce the nonlinear distortion.

**Figure 3:**
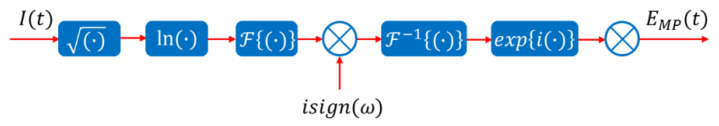
Signal processing of the KK receiver scheme.

Since the nonlinear square root operation and logarithmic operation of the KK algorithm will broaden the frequency spectrum of the signal, the signal is up-sampled before the KK algorithm processing, and the sampling rate is generally set to 4 [7]. Then, the KK receiver algorithm is used to recover the amplitude and phase of the signal, and the detail is shown in Figure 3. Due to the frequency interval between the carrier and the signal, the signal is an intermediate frequency signal at this time, and it needs to be down-converted to demodulate the baseband signal. To mitigate the inter-symbol interference caused by the limited bandwidth, a feedforward equalizer (FFE) is applied. However, the FFE boosts the in-band noise, which reduces the in-band signal-to-noise ratio (SNR). Therefore, a maximum likelihood sequence decision (MLSD) equalizer consisting of a post-filter and a conversional MLSD is employed. The post-filter is a two-tap finite impulse response filter. In addition, post-dispersion compensation is used in DSP for the 80 km SSMF transmission. After recovering the optical field with a KK receiver, the time domain information of the signal is transformed to the frequency domain by the fast Fourier transform (FFT) technique, and then the phase shift due to dispersion is converted back. Finally, the signal is converted to the time domain by the inverse fast Fourier transform (IFFT) technique.

## 4. Results and Discussions

**Figure 4:**
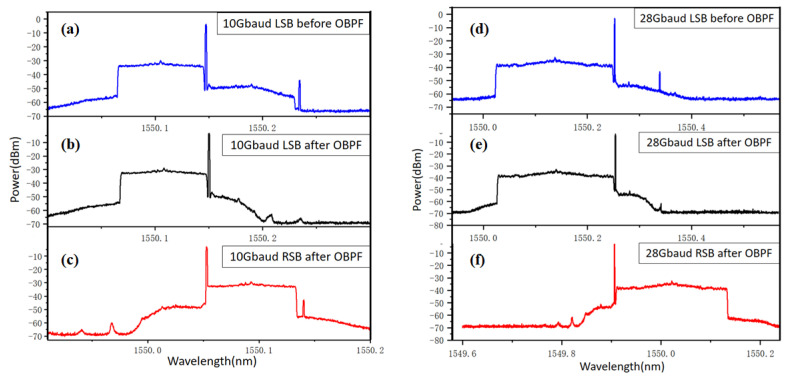
Optical spectra of the 10 Gbaud and 28 Gbaud QAM16 signals. (**a**) 10 Gbaud LSB signal before OBPF; (**b**) 10 Gbaud LSB signal after OBPF; (**c**) 10 Gbaud RSB signal after OBPF; (**d**) 28 Gbaud LSB signal before OBPF; (**e**) 28 Gbaud LSB signal after OBPF; (**f**) 28 Gbaud RSB signal after OBPF.

At a launch power of 11 dBm, corresponding to a CSPR of 13 dB, SBS can be easily observed. Figure 4 shows the optical spectra of the 10 Gbaud and 28 Gbaud QAM16 SSB signals after the 80 km SSMF transmission. From Figure 4a,d, one can clearly find the Stokes component induced by SBS at 1550.24 nm and 1550.27 nm for 10 Gbaud and 28 Gbaud QAM16, respectively, which exactly corresponds to an SBS frequency shift of 10.8 GHz with respect to the carrier wavelength. The anti-Stokes component will lead to attenuation at the shorter wavelength side with the same frequency shift. At 10 Gbaud, the anti-Stokes component is outside of the signal. The anti-Stokes component is completely buried in the LSB signal at 28 Gbaud. Therefore, it is unable to be observed from the spectra.

Using a flattop OBPF with high roll-off, one can mitigate the SBS induced nonlinear distortion by removing the Stokes component from the signal, as shown in Figure 4b,e for LSB signals. As for the RSB signal at a lower speed, such as 10 Gbaud in Figure 4c, the Stokes component would be too close to the signal and, thus, cannot be filtered out. As for the RSB signal at higher speeds, such as 28 Gbaud in Figure 4f, the Stokes component is also completely buried in the signal and, thus, cannot be filtered out either. Therefore, it can be expected that LSB suffers less SBS nonlinear distortion compared with RSB after the OBPF filtering.

**Figure 5:**
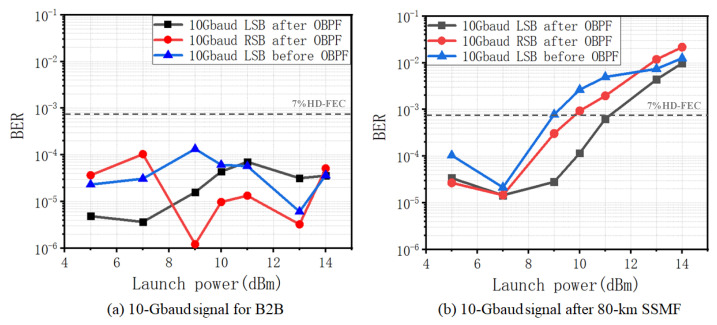
BER versus launch power. (**a**) 10 Gbaud for B2B; (**b**) after 80 km SSMF transmission.

Figure 5 shows the BER of the 10 Gbaud signal for back-to-back (B2B) and after 80 km SSMF transmission. One can observe from Figure 5a that the BER performances are at the same level and vary within a very small range along with the increase in the launch power for B2B. This is reasonable, since there is not yet any nonlinear distortion, and the CSPR is already large enough for the KK receiver to obtain an optimal BER. However, in Figure 5b, after 80 km SSMF transmission, significantly increased BER can be observed when the launch power exceeds ~7 dBm, which is mainly due to the existence of SBS. This is due to several reasons. The first is that the increased launch power is purely from the carrier from ECL2, with signal power remaining unchanged for the branch of ECL1 in Figure 2. Thus, CSPR increases accordingly, which should lead to reduced BER without nonlinear distortion. Secondly, signal power remains unchanged, which means that the nonlinear distortion due to Kerr effect by the signal is also maintained. Thirdly, the increase in BER is mainly induced by the increased carrier power, which can only be explained by the SBS induced nonlinear distortion.

As previously discussed, the nonlinear distortion caused by SBS can be mitigated with the use of OBPF. The conclusion can also be proved by the BER results in Figure 5b, which presents reduced BER for the signals after OBPF. Particularly, one can also observe reduced BER for LSB with respect to RSB, which is in good accordance with the principle. There is a ~2 dB power tolerance improvement for LSB with OBPF filtering with respect to the error-free BER of 3.8 × 10³.

**Figure 6:**
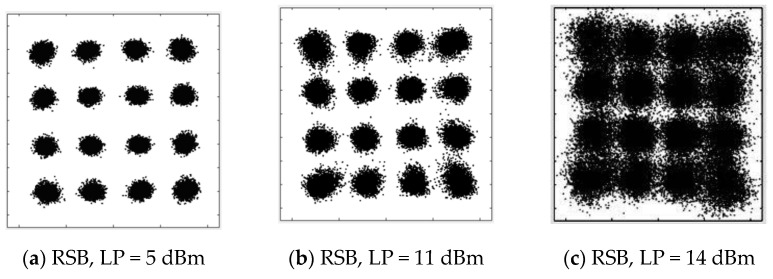

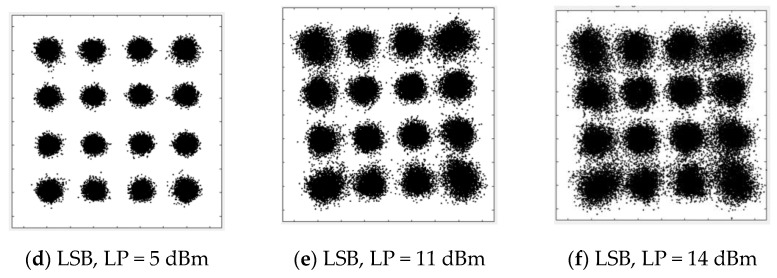
Constellation of 10 Gbaud QAM16 after 80 km SSMF transmission. (**a**) RSB, LP = 5 dBm; (**b**) RSB, LP = 11 dBm; (**c**) RSB, LP = 14 dBm; (**d**) LSB, LP = 5 dBm; (**e**) LSB, LP = 11 dBm; (**f**) LSB, LP = 14 dBm.

The nonlinear distortion can also be observed from the constellations, as depicted in Figure 6, at different launch powers of 5 dBm, 11 dBm, and 14 dBm. For both RSB and LSB, significantly increased noise rather than constellation shape distortion can be found in Figure 6b,c,e,f. This is also a proof of SBS rather than Kerr for the nonlinear distortion. Meanwhile, one can also observe slightly improved constellation noise performance for LSB at 14 dBm compared with that of RSB, which is in good agreement with previous predictions as well as the BER results.

**Figure 7:**
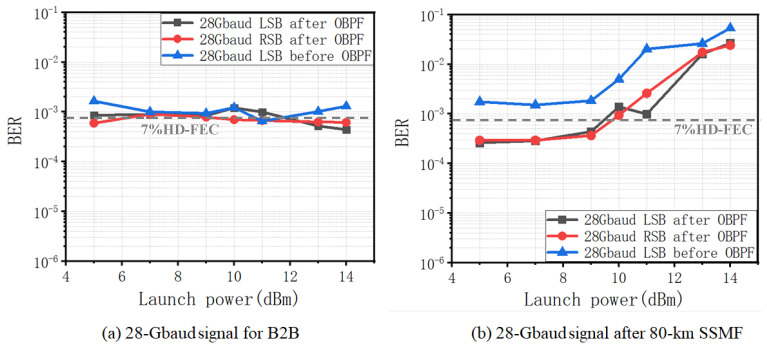
BER versus launch power. (**a**) 28 Gbaud for B2B; (**b**) after 80 km SSMF transmission.

**Figure 8:**
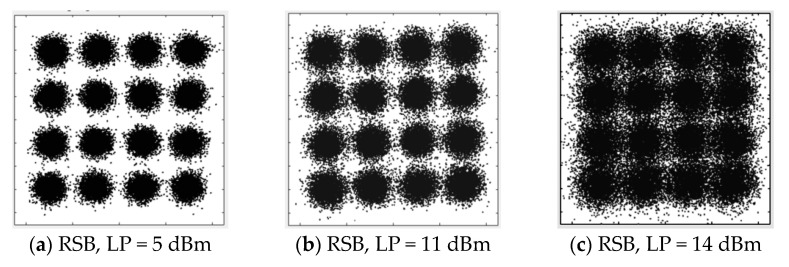

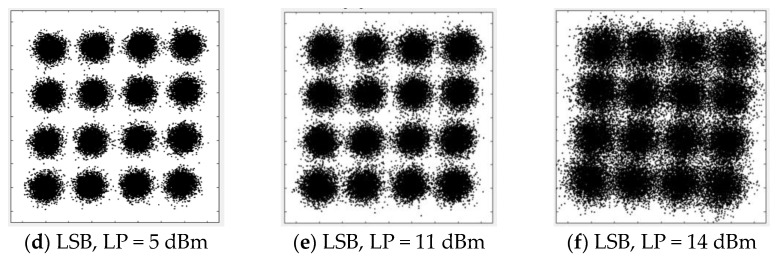
Constellation of 28 Gbaud QAM16 at 80 km SSMF transmission. (**a**) RSB, LP = 5 dBm; (**b**) RSB, LP = 11 dBm; (**c**) RSB, LP = 14 dBm; (**d**) LSB, LP = 5 dBm; (**e**) LSB, LP = 11 dBm; (**f**) LSB, LP = 14 dBm.

Similar performance can be observed for SSB signals at the 28 Gbaud data rate. The BER curves are shown in Figure 7. As for the B2B case in Figure 7a, BERs are maintained at almost the same level, since the CSPR is large enough with the absence of nonlinear distortion. The increased BER can be found in Figure 7b when the launch power exceeds 7 dBm, which is the same as that of the 10 Gbaud signals. This is also reasonable, since the threshold of the SBS should be the same for different data rates, which is one more proof of the SBS nonlinear distortion. The mitigation of the SBS nonlinear distortion can also be achieved by using the OBPF filtering with reduced BER for both LSB and RSB. However, there is no significant difference between LSB and RSB. We believe that this is due to the bandwidth limitation of the signal channel for 28 Gbaud, which to a certain extent erases their performance difference. This can also be observed from the performance of the constellations, as shown in Figure 8, in which the noise difference between LSB and RSB at 14 dBm is much smaller for 28 Gbaud than that for 10 Gbaud.

On the whole, SBS has different impacts on signals with varying data rates, and some of the impacts of the nonlinear distortion by SBS as CSPR increased can be alleviated by utilizing OBPF to filter out the component of SBS. The SBS has a greater overall impact on the higher data rate transmission systems than it does on the lower data rate systems when OBPF can be applied to filter out peak of SBS. For the higher rate transmission system, the impact of SBS on the RSB signal is greater than that on the LSB signal under the same experimental conditions.

## 5. Conclusions

In this work, the SBS induced nonlinear distortion for 10 Gbaud and 28 Gbaud SSB QAM16 transmission over an 80 km SSMF based on a KK receiver is experimentally observed and investigated for the first time. Significantly reduced BER can be induced along with the increase in CSPR due to SBS, which makes SBS an unneglectable issue for KK receiver-based systems. An SBS threshold of about 7 dBm is detected for such an 80 km SSMF link. Different nonlinear distortion performances are found for LSB and RSB signals, and LSB performs better than RSB. Here, a ~2 dB power tolerance improvement is achieved by LSB with optical filtering to remove the SBS. The proof-of-concept results in this work unfold an important issue of SBS-induced nonlinear distortion, which would be of great significance for KK receiver-based optical transmission applications.

## Data Availability

Not applicable.
